# Detection of Circulating SARS-CoV-2 Variants of Concern (VOCs) Using a Multiallelic Spectral Genotyping Assay

**DOI:** 10.3390/life13020304

**Published:** 2023-01-21

**Authors:** Andreas C. Chrysostomou, Antonia Aristokleous, Johana Hezka Rodosthenous, Christina Christodoulou, Georgia Stathi, Leondios G. Kostrikis

**Affiliations:** 1 Department of Biological Sciences, University of Cyprus, Aglantzia, 2109 Nicosia, Cyprus; 2 Cyprus Academy of Sciences, Letters, and Arts, 60-68 Phaneromenis Street, 1011 Nicosia, Cyprus

**Keywords:** COVID-19, SARS-CoV-2, variants of concern, molecular beacons

## Abstract

Throughout the coronavirus disease 2019 (COVID-19) pandemic, severe acute respiratory syndrome coronavirus 2 (SARS-CoV-2) has continuously evolved, resulting in new variants, some of which possess increased infectivity, immune evasion, and virulence. Such variants have been denoted by the World Health Organization as variants of concern (VOC) because they have resulted in an increased number of cases, posing a strong risk to public health. Thus far, five VOCs have been designated, Alpha (B.1.1.7), Beta (B.1.351), Gamma (P.1), Delta (B.1.617.2), and Omicron (B.1.1.529), including their sublineages. Next-generation sequencing (NGS) can produce a significant amount of information facilitating the study of variants; however, NGS is time-consuming and costly and not efficient during outbreaks, when rapid identification of VOCs is urgently needed. In such periods, there is a need for fast and accurate methods, such as real-time reverse transcription PCR in combination with probes, which can be used for monitoring and screening of the population for these variants. Thus, we developed a molecular beacon-based real-time RT-PCR assay according to the principles of spectral genotyping. This assay employs five molecular beacons that target ORF1a:ΔS3675/G3676/F3677, S:ΔH69/V70, S:ΔE156/F157, S:ΔΝ211, S:ins214EPE, and S:ΔL242/A243/L244, deletions and an insertion found in SARS-CoV-2 VOCs. This assay targets deletions/insertions because they inherently provide higher discrimination capacity. Here, the design process of the molecular beacon-based real-time RT-PCR assay for detection and discrimination of SARS-CoV-2 is presented, and experimental testing using SARS-CoV-2 VOC samples from reference strains (cultured virus) and clinical patient samples (nasopharyngeal samples), which have been previously classified using NGS, were evaluated. Based on the results, it was shown that all molecular beacons can be used under the same real-time RT-PCR conditions, consequently improving the time and cost efficiency of the assay. Furthermore, this assay was able to confirm the genotype of each of the tested samples from various VOCs, thereby constituting an accurate and reliable method for VOC detection and discrimination. Overall, this assay is a valuable tool that can be used for screening and monitoring the population for VOCs or other emerging variants, contributing to limiting their spread and protecting public health.

## 1. Introduction

Severe acute respiratory syndrome coronavirus 2 (SARS-CoV-2), which is the causative agent of coronavirus disease 2019 (COVID-19), emerged in December 2019 in Wuhan, China [1]. SARS-CoV-2 quickly began to spread around the world, and the World Health Organization (WHO) declared it a pandemic by March 2020 [2]. As of December 2022, SARS-CoV-2 has been responsible for approximately 650.7 million cases and 6.7 million deaths worldwide [3,4], which is a testament to the overwhelmingly high transmission and infection capabilities of the virus.

**Figure 1:**
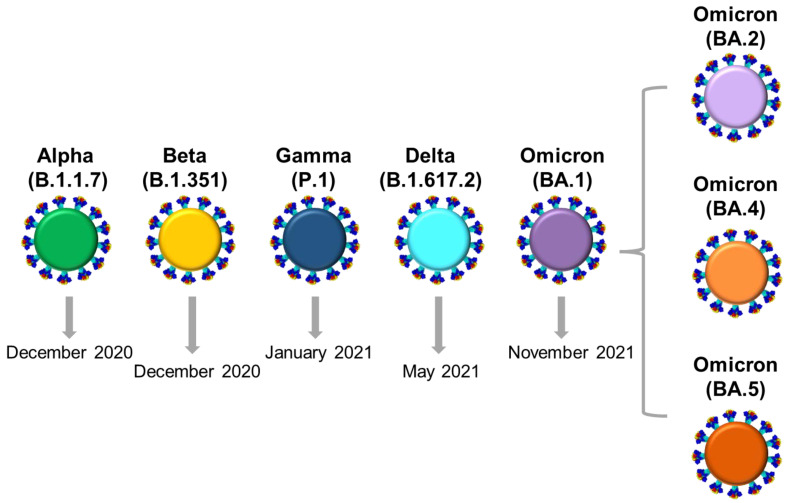
A schematic representation of SARS-CoV-2 variants of concern (VOCs). The VOC classification according to the WHO (The World Health Organization) nomenclature, as well as the Pango lineage, are written above each virion. Virions of Alpha (B.1.1.7) (green), Beta (B.1.351) (yellow), Gamma (P.1) (dark blue), Delta (B.1.617.2) (light blue), and BA.1 sublineage of the Omicron (B.1.1.529) VOC/lineages (purple) are shown [39]. The gray arrows indicate the month of the VOC designation by the WHO [13]. The gray bracket shows other sublineages of Omicron (B.1.1.529) in addition to BA.1 that have been prevalent: BA.2 (dark pink), BA.4 (orange), and BA.5 (dark orange) [40]. The illustration of the spike proteins on the colored virions underneath the names of each VOC/lineage, as also seen in figures below, was produced by PyMol (Version 2.4.1, Schrödinger, LLC, https://www.pymol.org, accessed on 18 February 2021) and is based on data derived and adapted from Protein Data Bank entry 6XEY [41,42], as well as other sources used to outline spike protein domains [43,44,45,46,47,48,49,50,51,52].

**Figure 2:**
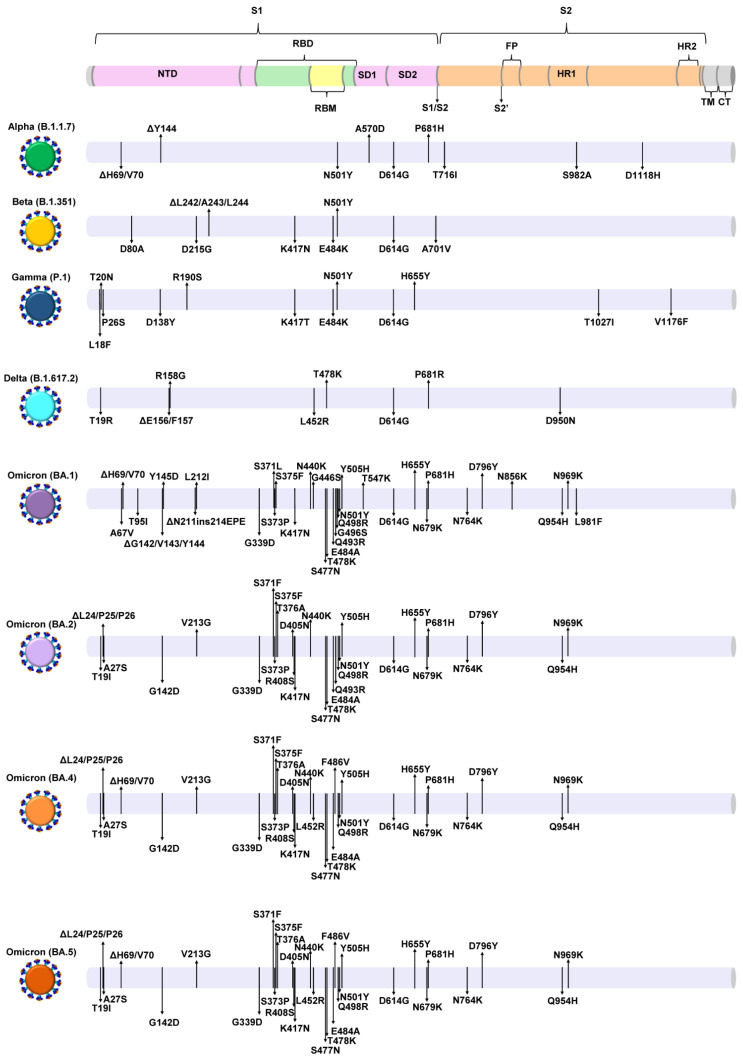
A graphical depiction of S protein mutations of SARS-CoV-2 VOCs. The figure shows the Alpha (B.1.1.7), Beta (B.1.351), Gamma (P.1), Delta (B.1.617.2), and Omicron (BA.1, BA.2, BA.4, BA.5) VOCs/lineages (colored virions). The colored cylinder on the top part of the figure shows the domains of the S protein based on Wuhan-Hu-1 [43,44,45,46,47,48,49,50,51,52]. The gray cylinders show the mutations found in different SARS-CoV-2 VOCs, as indicated with black arrows. The mutations indicated in the S protein of each VOC were derived from data provided by the WHO (The World Health Organization) [13].

The sustained transmission and replication of SARS-CoV-2, coupled with evolutionary pressures, such as the hosts’ immune system, antivirals, and vaccination, have contributed to the continuous evolution of this virus, the accumulation of mutations, and the differentiation of SARS-CoV-2 viral genomes into new variants [5,6,7,8,9,10,11,12,13,14]. In fact, the WHO has denoted certain SARS-CoV-2 viral genomes that have emerged over the course of the pandemic as variants of concern (VOCs) due to the risk they pose to public health as a result of their increased infectivity, immune evasion, and virulence (Figure 1 and Figure 2) [13,14,15]. Five VOCs have been denoted thus far, Alpha (WHO Greek alphabet nomenclature) (B.1.1.7, Pango classification system), Beta (B.1.351), Gamma (P.1), Delta (B.1.617.2), and Omicron (B.1.1.529), including their sublineages (Figure 1 and Figure 2) [13,16]. These VOCs have led to new outbreaks and waves, with massive surges of new SARS-CoV-2 infections, which have prompted global screening efforts for their identification and monitoring [17,18,19].

The most detailed method to identify such variants and the mutations they encompass is next-generation sequencing (NGS) [18,20,21,22]. Although NGS can yield a large amount of information, it requires a greater amount of time to yield results than methods based on polymerase chain reaction (PCR), and, as such, it is not an ideal approach during outbreaks caused by an emerging variant or when the incidence of infection is high [18,20]. A more suitable PCR-based method that allows for detection and tracking of variants such as VOCs is real-time reverse transcription PCR (real-time RT-PCR), especially when combined with probes, such as molecular beacons, as it yields results in a short amount of time [18,19,23,24,25]. Additionally, this method is accessible to the majority of laboratories around the world, especially in resource-limited settings, because it is not costly to implement [20].

Consequently, we developed a molecular beacon-based real-time RT-PCR method to detect and discriminate between VOCs according to an adaptation of the principles of spectral genotyping [26]. This assay employs a combinatorial pattern-based reporting system to achieve discrimination by using multiple molecular beacons at the same time, each targeting different deletions/insertion, found in different genome locations of SARS-CoV-2. The reason for targeting deletions and an insertion for this assay was owing to the high discriminatory power they inherently confer as a result of the larger nucleotide difference between genomes with and without the deletions/insertion [27,28]. Specifically, the molecular beacons designed for this assay target five deletions and one insertion involving the *ORF1a* and *S* genes of SARS-CoV-2 VOCs (ORF1a:ΔS3675/G3676/F3677, S:ΔH69/V70, S:ΔE156/F157, S:ΔN211, S:ins214EPE, and S:ΔL242/A243/L244). Targeting such mutations is important because they reportedly confer increased infectivity and immune evasion [29,30,31,32,33]. Additionally, ORF1a:ΔS3675/G3676/F3677 and S:ΔH69/V70 have been found in several VOCs [34,35] (Figure 2).

Moreover, molecular beacons were selected as probes due to the high specificity they offer and the extensive expertise and experience of our laboratory in molecular beacon technology [23,24,25,26,36,37]. Molecular beacons comprise a loop section (the target recognition sequence), a stem composed of two short sequences complementary to each other, a fluorescent dye covalently attached at the 5′-end, and a quencher attached at the 3′-end [23,24]. As a result of this structure, molecular beacons remain closed in the absence of their target but open, hybridize, and fluoresce when the target is present. Molecular beacons are therefore characterized by higher specificity in contrast to linear probes that do not require the thermodynamically costly dissociation of a stem to bind to their target [23,24,25,38]. Hence, molecular beacons are ideal tools for the detection and discrimination purposes required for this assay.

Herein, the development of a molecular beacon-based real-time RT-PCR assay for detection and discrimination of SARS-CoV-2 is explained from the design process to testing using SARS-CoV-2 samples from reference strains (cultured virus) and clinical patient samples. Using this assay, effective and rapid screening of the population for SARS-CoV-2 VOCs can be achieved to track these variants and stop their spread, helping to safeguard public health.

## 2. Materials and Methods

### 2.1. Molecular Beacon and Primer Design

**Figure 3:**
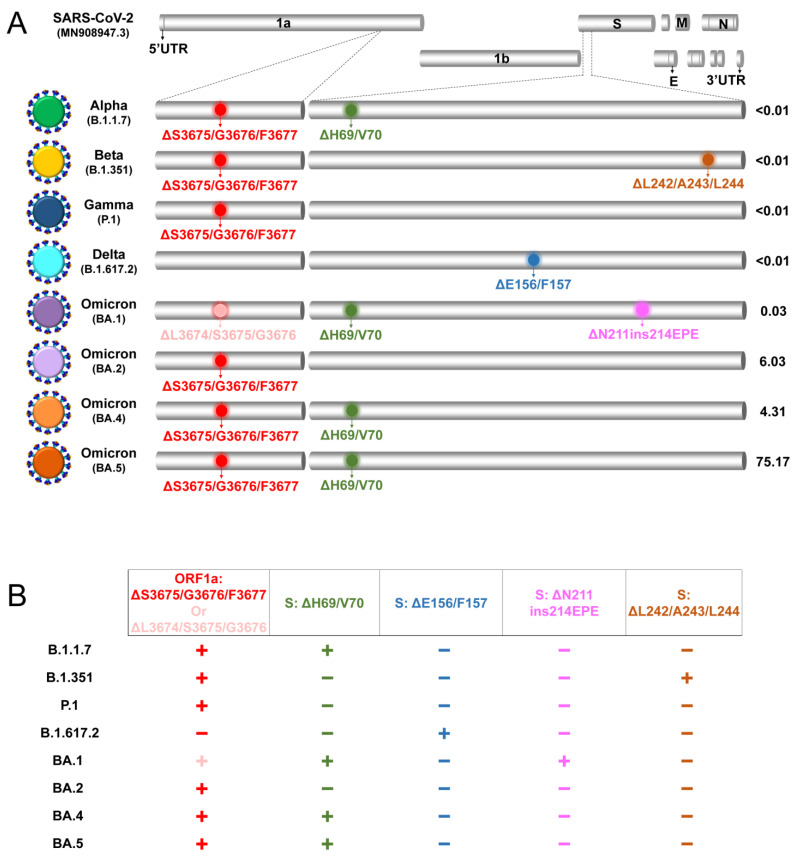
The deletion and insertion profiles of SARS-CoV-2 variant lineages focusing on the ORF1a and S proteins. (**A**) The genomic organization of SARS-CoV-2. Small regions of 75 amino acids (aa) (3645-3720 aa of ORF1a protein numbering) and 220 aa (50–270 aa of S protein numbering) are isolated using dotted lines. The Alpha (B.1.1.7), Beta (B.1.351), Gamma (P.1), Delta (B.1.617.2), and Omicron (BA.1, BA.2, BA.4, and BA.5) and their sublineages (variants of concern, VOCs) are depicted in gray cylinders along with the locations of the ORF1a:ΔS3675/G3676/F3677, S:ΔH69/V70, S:ΔE156/F157, S:ΔN211, and S:ΔL242/A243/L244 deletions and one insertion S:ins214EPE. The worldwide overall prevalence (%) for the period of 10 October to 7 November 2022 is depicted next to each cylinder and was identified using the CoV-Spectrum website (https://cov-spectrum.org/, date accessed 14 November 2022) [53]. (**B**) The S protein deletion and insertion profiles of the Alpha (B.1.1.7), Beta (B.1.351), Gamma (P.1), Delta (B.1.617.2), and Omicron (BA.1, BA.2, BA.4, and BA.5) and their sublineages are depicted. The presence of deletions/insertion is denoted by the symbol (+); absence is denoted by the symbol (−). Variant lineages were selected in accordance with their global public health significance as denoted by the World Health Organization.

The molecular beacons and primers used for this assay were created de novo (Table 1). The assay focused on the *ORF1a* and *S* gene regions of SARS-CoV-2 VOCs: Alpha (B.1.1.7), Beta (B.1.351), Gamma (P.1), Delta (B.1.617.2), and Omicron (BA.1, BA.2, BA.4, BA.5) (BA.3 had never risen to prevalence [40,53,54]). These regions contain the targets of the molecular beacons: the deletions ORF1a:ΔS3675/G3676/F3677, S:ΔH69/V70, S:ΔE156/F157, S:ΔΝ211, and S:ΔL242/A243/L244 as well as the insertion S:ins214EPE, with each of them reported to be highly prevalent in specific VOCs (Figure 3) [13,53,55]. ORF1a:ΔS3675/G3676/F3677 is a set of three amino acid (aa) deletions found in Alpha, Beta, Gamma, and Omicron (BA.2, BA.4, BA.5). S:ΔH69/V70 is a two-aa deletion in Alpha and Omicron (BA.1, BA.4 and BA.5). S:ΔE156/F157 involves a two-aa deletion in Delta (also reported as S:ΔF157/R158 [56,57]). S:ΔΝ211 and S:ins214EPE are a one-aa deletion and a three-aa insertion in Omicron (BA.1), respectively. S:ΔL242/A243/L244 involves a three-aa deletion in Beta (also reported as S:ΔL241/L242/A243 [58]) (Figure 3) [13,53,55]. It is important to note that selecting deletions/insertion as targets was crucial for the purposes of this assay, as they introduce a larger nucleotide difference in contrast to single-nucleotide polymorphisms (SNPs), thereby increasing the discriminatory capacity of the assay [27,28].

The identification and isolation of suitable SARS-CoV-2 genomic regions containing the abovementioned deletions/insertion was performed through the generation of multiple sequence alignments (MSAs) using sequences downloaded from GISAID’s database [59] (accessed on 5 July 2021 and 29 November 2021) with respect to each VOC. Additionally, the reference genome Wuhan-Hu-1 (GenBank accession number MN908947.3) [60,61] was included in the MSAs, which were performed using MAFFT web server version 7 (https://mafft.cbrc.jp/alignment/server/ accessed on 5 July 2021 and 29 November 2021) [62,63] and viewed with the AliView program version 1.26 [64]. The primers and molecular beacons used in this assay were designed based on the identification of these regions.

Each primer was designed to have a high GC content to ensure binding to its target, and each primer pair amplifies small regions surrounding the target. These target amplicons encompassing SARS-CoV-2 deletions/insertion are 99–153 nucleotides (Table 1). The melting temperature (Tm) of the primers was calculated using the Integrated DNA Technologies, Inc. (IDT) Oligo Analyzer tool (Integrated DNA Technologies, Inc., Coralville, IA, USA) (https://eu.idtdna.com/pages last accessed on 2 December 2021).

**Figure 4:**
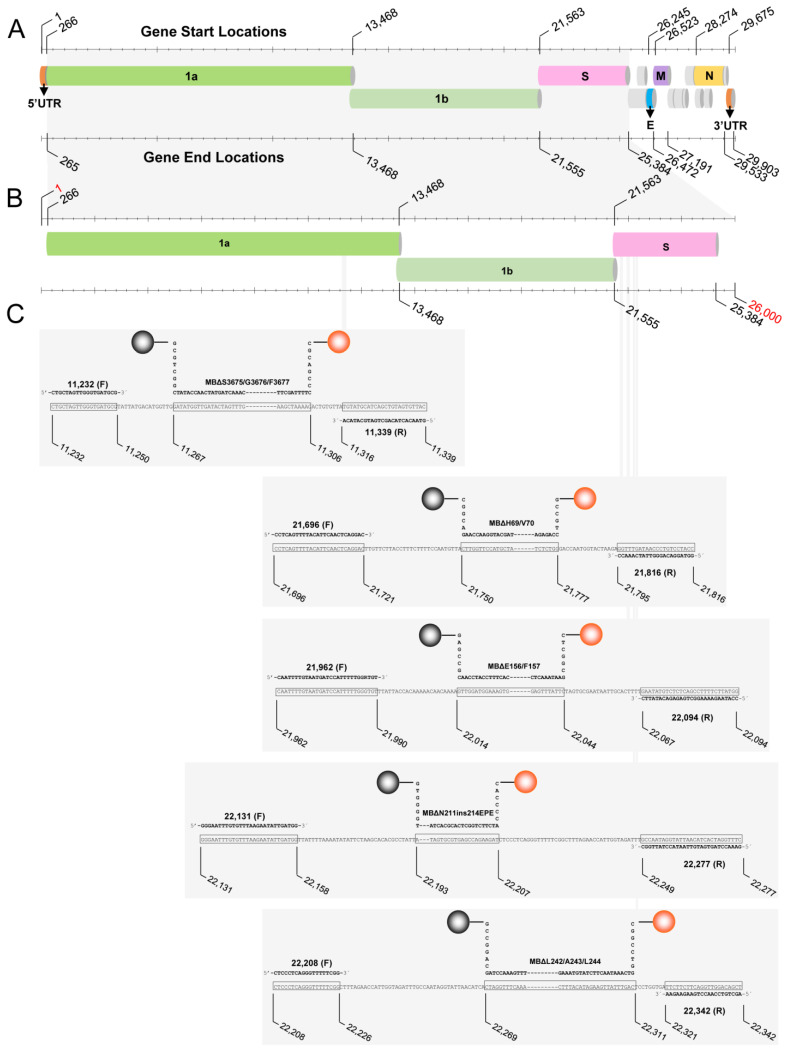
Graphic illustration of the genome of SARS-CoV-2 and the regions targeted by the assay. (**A**) The regions targeted by the assay are indicated in the highlighted area. (**B**) The start and end locations of these regions are demonstrated by the nucleotide positions in the visually amplified area. (**C**) Target regions are further magnified to show the amplicons that include the five deletions and one insertion (ORF1a:ΔS3675/G3676/F3677, S:ΔH69/V70, S:ΔE156/F157, S:ΔN211, S:ins214EPE, and S:ΔL242/A243/L244). The sequences of primers and molecular beacons are shown in bold, and their targets are shown in black boxes. The numbers shown for the probes and primers are based on the SARS-CoV-2 reference genome Wuhan-Hu-1 (GenBank: MN908947.3). The gray spheres (left side of each beacon) indicate the quencher DABCYL (N-[4-(4-dimethylamino) phenylazo] benzoic acid) at the 3′-end of the molecular beacon; the orange spheres (right side of each beacon) indicate the fluorophore HEX (N-HEX-6-aminohexanol) beacon at the 5′-end.

Each molecular beacon was designed such that the stems were complementary to each other, five to seven nucleotides long, and had a high GC content (Table 1, Figure 4). All molecular beacons were labeled with N-HEX-6-aminohexanol (HEX) fluorophore at the 5′-end and N-[4-(4-dimethylamino) phenylazo] benzoic acid (DABCYL) at the 3′-end. The loop section of the molecular beacons that contains the sequence complementary to the target is 21-34 nucleotides long and high in GC content (Table 1, Figure 4). Once each molecular beacon was designed, its folding properties and structure along with the thermodynamic details were examined using the Mfold server for nucleic acid folding and hybridization prediction (http://www.unafold.org/mfold/applications/dna-folding-form.php, last accessed on 2 December 2021) [65]. These design steps were taken to ensure that the molecular beacons only open in the presence of their target while retaining a closed conformation in its absence. In addition to designing molecular beacons and primers, short oligonucleotide sequences complementary to the loop section of each molecular beacon, termed correct targets, were designed to assess the behavior of the molecular beacons in the presence of their targets. Furthermore, oligonucleotides termed incorrect targets were designed to examine the behavior of the molecular beacons in the presence of a non-fully complementary SARS-CoV-2 target. Specifically, these incorrect targets are based on Wuhan-Hu-1 and include the relative regions of the genome targeted by the molecular beacons; however, as this reference genome is classified as B lineage [12], it does not contain any of the deletions/insertion found in the five VOCs targeted by the molecular beacons. Moreover, we provide the option of an internal positive control (IPC) designed using sections of the *S*, *E*, *M*, and *N* genes of the SARS-CoV-2 reference genome Wuhan-Hu-1 (MN908947.3) (explained in detail in [36]).

The design process was concluded by examining the thermodynamic compatibilities of the molecular beacons with the primers and targets (correct and incorrect). This step ensured that no primer self-dimers or heterodimers formed and that the Tm of the molecular beacon–correct target complex was higher than the molecular beacon–incorrect target complex, such that the molecular beacon bound with its correct target more strongly than with the incorrect target. The molecular beacons, primers, and targets were synthesized by Biosearch Technologies (Risskov, Denmark).

### 2.2. Thermal Profiles of Molecular Beacons

Melting curve analysis was performed using a 7900HT Fast Real-Time PCR System (Applied Biosystems, Foster City, CA, USA) to assess the thermodynamic characteristics of the molecular beacons. The molecular beacons were tested with no target in the presence of the correct target and the incorrect target. The final reaction volume of the molecular beacon with no target was 25 μL and consisted of 5 μL of 4× TaqPath™ 1-Step Multiplex Master Mix (No ROX) (Life Technologies, Frederick, MD, USA), 3 μL (5 pmol/μL) of molecular beacon, and 17 μL of nuclease-free H_2_O. The reactions with the target (correct or incorrect) also contained 1 μL of target (100 pmol/μL) (correct/incorrect) and 16 μL of nuclease-free H_2_O (instead of 17 μL). The PCR cycling conditions consisted of 1 cycle for 2 min at 95 °C, followed by 50 cycles split into two steps: the first step, during which fluorescence data were collected, was at 80 °C for 30 s, decreasing by 1 °C per cycle; the second step was at 80 °C for 10 s, decreasing by 1 °C per cycle. Fluorescence was measured at 535 nm for HEX and was recorded at each cycle. Following the completion of the run, the fluorescence signal data were normalized and plotted.

### 2.3. Real-Time RT-PCR

Real-time RT-PCR was performed using a 7900HT Fast Real-Time PCR System (Applied Biosystems, Foster City, CA, USA) with 4× TaqPath™ 1-Step Multiplex Master Mix (No ROX) (Life Technologies, Frederick, MD, USA). Each 30 μL reaction consisted of 5 μL of RNA, 7.5 μL of 4× TaqPath™ 1-Step Multiplex Master Mix, 1.5 μL of 20 pmol/μL of each primer, 3.0 μL of 5 pmol/μL molecular beacon, and 11.5 μL of nuclease-free H_2_O. The primers and molecular beacons used are shown in Table 1. The reverse transcription cycling conditions consisted of 1 cycle at 25 °C for 2 min, followed by 1 cycle at 53 °C for 10 min and 1 cycle at 95 °C for 2 min. This was followed by 5 cycles at 95 °C for 3 s and 53 °C for 30 s. Finally, 35 cycles at 95 °C for 3 s and 53 °C for 30 s were performed, during which data collection occurred. In these runs, no-template controls (NTCs) (nuclease free H_2_O) and SARS-CoV-2 RNA BetaCoV/Germany/BavPat1/2020 p.1” grown in cell culture, B.1 lineage (EVAg, Charité, Berlin, Germany) or UVE/SARS-CoV-2/2020/FR/702 (MT777677.1) (EVAg, Charité, Berlin, Germany) were used as negative controls. A sample was considered positive when the fluorescence signal exceeded the threshold line before the 40th cycle. Following the completion of the run, the fluorescence signal data were plotted.

### 2.4. Reference and Clinical Samples Used to Test the Assay

The assay was tested using a panel of reference samples derived from cultured SARS-CoV-2 virus (Vero E6 cells) obtained from European Virus Archive goes Global (EVAg, Charité, Berlin, Germany) (Table 2). These samples correspond to the first four VOCs, Alpha (B.1.1.7), Beta (B.1.351), Gamma (P.1), and Delta (B.1.617.2). Additionally, a fifth sample of the B.1 lineage was received and used as a negative control during the real-time RT-PCRs performed with these reference samples because it does not contain any of the deletions/insertion targeted by this assay. No Omicron, a VOC that emerged at a later date (November 2021), samples were available at the time of the order to EVAg (27 July 2021). The five reference samples were received by our laboratory, the Laboratory of Biotechnology and Molecular Virology of the University of Cyprus (BMV UCY), in lyophilized form and stored at −20 °C until processing.

In addition to the EVAg panel of reference samples, the assay was tested using clinical samples (Table 2) that were derived from the study of the Genomic Epidemiology of the SARS-CoV-2 Epidemic in Cyprus ([66] and manuscript in preparation for publication), which were collected under the ongoing collaboration between our laboratory (BMV UCY), the Ministry of Health, NIPD Genetics, and the other members of the Cypriot Comprehensive Molecular Epidemiological Study on SARS-CoV-2 (COMESSAR) Network. Bioethical approval for the use of these samples was granted by the Cyprus National Bioethics Committee (EEBK 21.1.04.43.01). To ensure patient anonymity, all samples received by BMV UCY were double coded to ensure that no connection between the samples and corresponding study subjects could be made. The collection and use of the samples were in accordance with the relevant guidelines and regulations of the Cyprus National Bioethics Committee.

These clinical samples were collected during the period of October 2021 to August 2022 by NIPD Genetics and processed at their facilities. The extraction of RNA from these nasopharyngeal samples, real-time RT-PCR, and NGS are explained in detail in our previous publication on the molecular epidemiology of Cyprus [67]. Our laboratory was initially provided with the near-full SARS-CoV-2 NGS sequences of these samples that were used for studying the genomic epidemiology of the SARS-CoV-2 epidemic in Cyprus, through which lineages were identified. Nasopharyngeal samples were subsequently received and used for the development of the present assay. These samples were stored at -80 °C upon arrival at BMV UCY until processing. The lineages of each of the clinical sample sequences were reconfirmed using Pangolin Webtool [68] (Pangolin-data version v1.14, Pangolin version 4.1.1, https://pangolin.cog-uk.io/, accessed on 7 September 2022) (Table 2). However, it is important to note that only samples of Delta and Omicron (including their sublineages) were available/stored by NIPD Genetics, and, as such, there were no clinical samples of the Alpha, Beta, and Gamma VOCs to be tested using this assay. Nonetheless, this was not considered an issue because these VOCs were covered by the panel of reference samples from EVAg.

Once the panel of reference samples and clinical samples were stored at BMV UCY, RNA extraction was performed using a QIAmp Viral RNA Mini Kit (Qiagen, Hilden, Germany) and the QIAcube Connect machine (Qiagen, Hilden, Germany) in accordance with the manufacturer’s specifications. For the panel of reference samples, which were in lyophilized form, this entailed reconstitution with 200 μL of sterile distilled water (per EVAg’s specifications). Thus, there was sufficient volume for only one RNA extraction because the volume requirement of the RNA extraction protocol is 140 μL of the initial sample. This volume limitation also applied to the clinical samples, precluding the use of samples with a volume less than 140 μL. Once the RNA extraction was completed, the assay was tested using the abovementioned real-time RT-PCR protocol (Section 2.3).

## 3. Results

### 3.1. Molecular Beacon Thermal Denaturation Profiles

**Figure 5:**
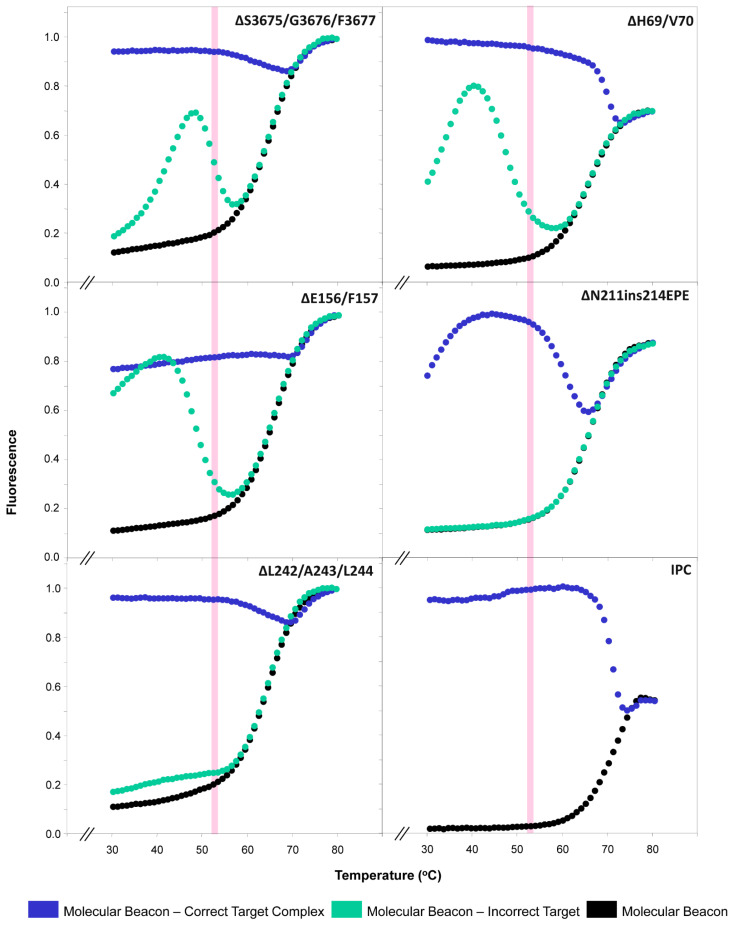
Thermal denaturation profiles of molecular beacons for the deletions/insertion ORF1a:ΔS3675/G3676/F3677, S:ΔH69/V70, S:ΔE156/F157, S:ΔN211ins214EPE, and S:ΔL242/A243/L244, and the internal positive control (IPC). Melting curve analysis was used to determine the thermal denaturation profiles of the molecular beacons. The figure demonstrates normalized fluorescence thermal transitions of molecular beacons: without target shown in black, beacon–correct target complexes shown in blue, and beacon–incorrect target interaction shown in turquoise. The y-axis represents the normalized fluorescence data; the x-axis represents the temperature. The pink-highlighted area represents the temperature that was selected for the discrimination between the molecular beacon–correct target complex versus the molecular beacon–incorrect target.

Following the melting curve analyses of each molecular beacon, the fluorescence for the beacon–target hybrids (correct and incorrect) and for the beacon without target was plotted against temperature to ascertain their thermal characteristics, as well as to identify the window of discrimination. The window of discrimination is denoted as the range of temperatures at which the complex of the molecular beacon with the correct target had the highest fluorescence difference compared to the molecular beacon with the incorrect target and the molecular beacon without the target. Once the window of discrimination was identified, an optimal temperature within it was selected and used as the annealing temperature for real-time RT-PCR. As shown in Figure 5, the mean melting temperature of the molecular beacons without a target was ~64.2 °C, which was approximately the same as the mean melting temperature (64 °C) estimated during the design part of the assay using Mfold [65]. The largest difference in fluorescence signal (the window of discrimination) between all molecular beacons without target and all molecular beacon-target (correct and incorrect) hybrids ranged from 45 to 60 °C, with the mean temperature within this window being 52.5 °C (Figure 5).

Thus, taking into account the melting temperatures of the beacons (~64.2 °C), the mean temperature of the windows of discrimination (52.5 °C), and the estimated mean melting temperature of the primers (~55 °C) using IDT, the temperature selected for the annealing stage of real-time RT-PCRs was 53 °C (Figure 3, pink highlighted area). This was further supported by the mean melting temperatures of the molecular beacon–correct and incorrect target hybrids, which were estimated to be ~55 °C and ~45 °C, respectively. The selected temperature of 53 °C not only coincides with our previous molecular beacon-based real-time RT-PCR assay [36] but also allows for all reactions to be performed during the same run, thereby improving the time and cost efficiency of the assay.

### 3.2. Real-Time RT-PCR Testing Results

**Figure 6:**
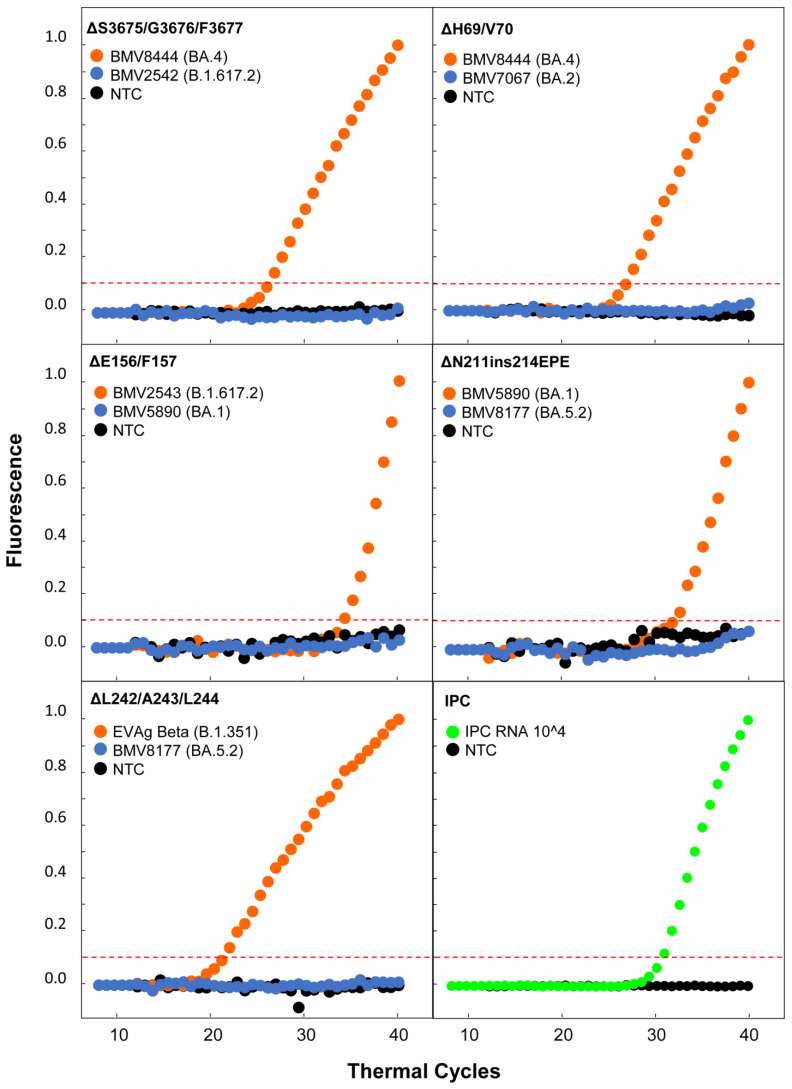
Uniplex real-time RT-PCR for the five different SARS-CoV-2 VOC deletions and one insertion (ORF1a:ΔS3675/G3676/F3677, S:ΔH69/V70, S:ΔE156/F157, S:ΔN211ins214EPE, and S:ΔL242/A243/L244) and the IPC. The normalized fluorescence signal graph is shown for the molecular beacons and their respective correct targets as well as an incorrect target. The correct target amplification curve is indicated with orange dots, the incorrect target amplification is indicated with blue dots, and the negative control (no-template control, NTC) is indicated with black dots. The y-axis represents fluorescence; the x-axis represents the thermal cycles. The intermittent red line indicates the threshold cycle.

The assay was tested using the reference and clinical samples presented and explained in Section 2.4. A representation of the performance of each molecular beacon with samples encompassing the correct and incorrect targets, as well as no target, is shown in Figure 6; the full list of the real-time RT-PCR testing results is provided in Table 2 and Table S1. Each molecular beacon was tested with every sample, and the pattern of positive “+” (presence of the targeted deletion/insertion) and negative “−” (absence of the targeted deletion/insertion) results enabled the discrimination of VOCs (Figure 3 and Table 2). As the negative controls of the B.1 lineage do not encompass any of the deletions/insertion found within the five VOCs, there was no fluorescence with any of the molecular beacons, as expected (Table 2). This pattern of full negative “−” results indicated that with this assay, these B.1 samples were correctly determined to not be any of the five VOCs. The reactions with the Alpha reference sample showed a positive “+” result with the molecular beacons targeting ORF1a:ΔS3675/G3676/F3677 and S:ΔH69/V70 and “−“ results with the other three molecular beacons. This pattern of results is in agreement with the expected results shown in Figure 3; therefore, this assay was able to identify the presence of the targeted deletions with respect to the Alpha VOC. It is important to note that the pattern of “+” and “−“ for Alpha is the same as that for Omicron BA.4 and BA.5 (and their sublineages), and this assay correctly identified the presence of ORF1a:ΔS3675/G3676/F3677 and S:ΔH69/V70 for all clinical samples classified as BA.4 and BA.5 (Table 2). However, Alpha is denoted as a previously circulating VOC by the WHO [13], and for the recent period of October–November 2022, Omicron BA.4 was significantly less represented than BA.5, with 4.31% and 75.17% worldwide prevalences, respectively (Figure 3) [53]. Similarly, the same correct pattern of results was found for Gamma and Omicron BA.2, with only ORF1a:ΔS3675/G3676/F3677 being identified, and none of the other four deletions/insertion were detected. Similar to Alpha, the Gamma VOC is denoted as a previously circulating VOC, but Omicron BA.2 is still in circulation (Figure 3, Table 2) [13]. Conversely, the Beta, Delta, and Omicron BA.1 reference and clinical samples displayed a distinct pattern of results, as a molecular beacon was designed to identify a unique target for each of their respective VOCs. Specifically, the unique target of the Beta VOC used was S:ΔL242/A243/L244, and the other target for Beta was ORF1a:ΔS3675/G3676/F3677, which, as seen above, is common to Alpha, Beta, Gamma, and Omicron (BA.2, BA.4, BA.5). The results of the Beta reference sample correctly indicated “+” for ORF1a:ΔS3675/G3676/F3677 and S:ΔL242/A243/L244, and the results for all other molecular beacons were “−“ (Figure 3, Table 2). The unique target for Delta was S:ΔE156/F157, and the results for all Delta reference and clinical samples were “+” only for this target and “−” for all others, as expected. The unique target for Omicron BA.1 was S:ΔN211ins214EPE, which includes a deletion and an insertion; the other target was S:ΔH69/V70, which is common to Alpha and Omicron (BA.1, BA.4, and BA.5). The results for all Omicron BA.1 clinical samples were correctly identified as “+” for both S:ΔH69/V70 and S:ΔN211ins214EPE. Additionally, for all Omicron BA.1 samples, the molecular beacon targeting ORF1a:ΔS3675/G3676/F3677 indicated “+”, even though this deletion is not common to Omicron BA.1 [55]. This is due to the molecular beacon identifying the three-aa deletion ORF1a:ΔL3674/S3675/G3676, which is common to Omicron BA.1 and is similar to ORF1a:ΔS3675/G3676/F3677 [35,55]. This shows that the molecular beacon MBΔS3675/G3676/F3677 was able to identify both ORF1a:ΔS3675/G3676/F3677 and ORF1a:ΔL3674/S3675/G3676, as genomes with these deletions only differ by one nucleotide at the region targeted by the molecular beacon [35]. Nonetheless, this did not impact the ability of the assay to detect and discriminate samples classified as Omicron BA.1. Notably, Omicron BA.1 has been de-escalated by the European Centre for Disease Prevention and Control (ECDC) and is detected at extremely low levels [40,53], and both the aforementioned Beta and Delta VOCs are denoted as previously circulating VOCs [13].

## 4. Discussion

Since the beginning of the COVID-19 pandemic, new variants have emerged, with a few of them being denoted as VOCs, given their high impact on public health [69]. These variants have often led to massive surges in SARS-CoV-2 infections, highlighting the urgent need for their quick identification and monitoring to stop their spread and safeguard public health [17,18,19]. The detection and discrimination of such variants during these outbreak periods can be accomplished through methods with a quick turnaround time, such as real-time RT-PCR, especially when combined with probes, such as molecular beacons [70]. In fact, molecular beacons are ideal for detecting mutations and discriminating variants because they are characterized by extraordinary sensitivity and specificity [23,24,25]. Moreover, they only require a short amount of time for design, synthesis, and testing, making them the perfect tool during high incidence, when speed is of utmost importance [38].

The molecular beacon-based real-time RT-PCR assay presented in this manuscript not only outlines a method for the detection and discrimination of VOCs but also, more importantly, presents an approach that can be applied to any emerging variant. The latter has already been showcased with the emergence of Omicron in November 2021 [71]. Specifically, this assay was originally designed to detect the first four VOCs, which, as shown in Table 2 and Figure 6, it accurately does so with high specificity, and was rapidly adapted with a molecular beacon designed to target the Omicron (BA.1) S:ΔN211 deletion and S:ins214EPE insertion, which are unique among VOCs [55]. Similarly, the results of this study showed that the molecular beacon targeting the S:ΔN211 S:ins214EPE insertion is highly specific and did not react with any other targets. In addition to ΔN211ins214EPE Omicron (BA.1), the deletions S:ΔE156/F157 (Delta) and S:ΔL242/A243/L244 (Beta), which are unique among VOCs, were targeted. Focusing on these deletions was important for the assay’s goal to be able to discriminate between VOCs; however, recurrent mutations were also targeted to increase the flexibility and applicability of the assay. This strategy of targeting recurrent mutations is important for the purposes of this assay as it allows for a molecular beacon to still be used in the case that a lineage falls out of circulation and another one encompassing the same mutation targeted by the beacon emerges. This exact scenario occurred with Alpha, Beta, and Gamma, for which a molecular beacon was designed to target the ORF1a:ΔS3675/G3676/F3677 recurrent deletion [13,35,53,55]. All three of these variants carrying this deletion are denoted as previously circulating VOCs by the WHO [13], yet ORF1a:ΔS3675/G3676/F3677 was identified again in Omicron BA.2, BA.4, and BA.5 [13,35,53,55]. Notably, Omicron BA.1 (de-escalated, low prevalence [40]) has a similar mutation, ORF1a:ΔL3674/S3675/G3676; the molecular beacon MBΔS3675/G3676/F3677 can also detect it, as genomes with these deletions only show a single-nucleotide difference in the target recognized by the beacon [35,55] (explained in Section 3.2). Although the molecular beacon for ORF1a:ΔS3675/G3676/F3677 was designed prior to the emergence of Omicron BA.1, the fact that ORF1a:ΔL3674/S3675/G3676 is also detected using this beacon can serve as a potential increase in the number of combinations for the pattern-based discrimination system used for this assay (Table 2). Similar to ORF1a:ΔS3675/G3676/F3677, the recurrent deletion S:ΔH69/V70 [34] was also used to increase the flexibility and applicability of the assay, and it is found in Alpha and Omicron (BA.1, BA.4 and BA.5). As noted above, only Omicron BA.2, BA.4, and BA.5 are currently circulating, with BA.5 constituting the overwhelming majority of infections (Figure 3) [53]. Thus, the strategy outlined for this assay shows how it can be employed for detection and discrimination of VOCs and also how it can be easily adapted or retain its relevance in the event of emergence and re-emergence of the targeted mutations.

The molecular beacons used in this assay displayed high specificity in identifying their intended target and discriminating against the incorrect target, as the results show that there was no fluorescence with incorrect targets (Figure 6). This can also be attributed to the decision to focus solely on deletions/insertion as targets for the molecular beacons, in contrast to other presently available assays [18,20,38,70,72,73], due to the high discrimination power that deletions/insertions inherently offer as targets, since they are characterized by at least a three-nucleotide difference [27,28]. This decision to focus on deletions/insertions stemmed from one of the scopes of the design of this assay, which was to promote this methodology to laboratories that intend to employ SARS-CoV-2 detection assays, even if they do not necessarily possess extensive experience in molecular beacon design. Despite the prowess of molecular beacons to detect only their target, even at a one-nucleotide difference [23,24,25], the intricacies of designing molecular beacons that can detect SNPs also pose the risk of false-negative results in the case that there is another mutation within the target recognition sequence [38]. Although this can certainly be solved by redesigning the molecular beacon [38], the probability of SARS-CoV-2 acquiring an SNP within the target sequence remains [9,15]. Nonetheless, the usefulness of targeting SNPs with molecular beacons is valuable, especially when designed with necessary expertise and precautions [26,38]; indeed, the combinations for the pattern-based discrimination nature of the assay can be expanded through the addition of these mutations as targets.

Despite the strengths of this assay listed above, it was limited by the number of clinical samples tested because certain VOCs were not available in storage. Regardless, validation of the assay can be performed by testing it with a significantly larger number of clinical samples with known lineages (through NGS) and concentrations, which was not provided for the clinical samples used in the present study. The latter can also provide the sensitivity and limit of detection of the assay. Alternatively, the limit of detection can be obtained by constructing standard curves using in vitro RNA transcripts, as explained in our previous publications of molecular beacon-based real-time RT-PCR assays [36,37]. Furthermore, the assay can be validated through external quality assessments and using blinded samples from Quality Control for Molecular Diagnostics (QCMD) or the WHO [36,74]. Taking these steps will provide the necessary basis for this assay to be implemented in clinical or commercial settings.

This study lays down the foundational work and presents the methodology, as well as the completed designs for primers and molecular beacons, for this assay, which can be extrapolated for private use, commercialization, and clinical testing. Importantly, this assay has immense potential for growth, as the repertoire of primers and molecular beacons (Table 1) can be exponentially expanded upon, with an increasingly higher number of combinations that can be used to detect future circulating variants; or even have an increased resolution to be able to discriminate between their sublineages. The presented strategy, methodology, and constructs of this assay are a valuable asset to any laboratory aiming to classify positive SARS-CoV-2 samples and discern the circulating VOCs in a region. Additionally, it can be assimilated into other already existing assays or even be adapted for the detection and discrimination of other pathogens. However, it is always important to exercise caution and examine the genetic variability of SARS-CoV-2 within the targeted regions of both the primers and molecular beacons to ensure that any accumulated mutations within those regions do not impede the functionality of the assay. One of the strengths of this assay is that it can be easily and swiftly adapted if the above scenario occurs.

In conclusion, we provide an accurate and reliable uniplex molecular beacon-based real-time RT-PCR assay that can be used for detection and discrimination of VOCs. This assay includes five molecular beacons designed to target ORF1a:ΔS3675/G3676/F3677, S:ΔH69/V70, S:ΔE156/F157, S:ΔN211ins214EPE, and S:ΔL242/A243/L244 deletions/insertion, and each separate reaction with these molecular beacons can be run under the same conditions, with cost and time efficiency. The assay is able to accurately identify the correct VOCs when tested using reference samples from cultured virus received from EVAg (Charité, Berlin, Germany), as well as clinical samples previously classified by NGS. Therefore, this assay can be used for rapid and accurate screening and monitoring of the population for SARS-CoV-2 to stop the spread of such variants, contributing to the protection of public health.

## Figures and Tables

**Table 1:** Oligonucleotide PCR primers, target amplicons, and molecular beacons used in the real-time RT-PCR assay.

Designation ^a^	Target Gene	Sequence	Position ^b^	Amplicon Length (nts) ^c^	Gene Accession Number ^d^	Reference ^e^
**PCR Primers**						
11,232 (F)	*ORF1a*	CTGCTAGTTGGGTGATGCG	11,232–11,250		MN908947.3	This study
11,339 (R)	*ORF1a*	GTAACACTACAGCTGATGCATACA	11,316–11,339		MN908947.3	This study
21,696 (F)	*S*	CCTCAGTTTTACATTCAACTCAGGAC	21,696–21,721		MN908947.3	This study
21,816 (R)	*S*	GGTAGGACAGGGTTATCAAACC	21,795–21,816		MN908947.3	This study
21,962 (F)	*S*	CAATTTTGTAATGATCCATTTTTGGRTGT	21,962–21,990		MN908947.3	This study
22,094 (R)	*S*	CCATAAGAAAAGGCTGAGAGACATATTC	22,067–22,094		MN908947.3	This study
22,131 (F)	*S*	GGGAATTTGTGTTTAAGAATATTGATGG	22,131–22,158		MN908947.3	[36]
22,208 (F)	*S*	CTCCCTCAGGGTTTTTCGG	22,208–22,226		MN908947.3	This study
22,277 (R)	*S*	GAAACCTAGTGATGTTAATACCTATTGGC	22,249–22,277		MN908947.3	[36]
22,342 (R)	*S*	AGCTGTCCAACCTGAAGAAGAA	22,321–22,342		MN908947.3	This study
26,355 (R)	*E*	AAGCGCAGTAAGGATGGCTA	26,336–26,355		MN908947.3	[36]
**Target Amplicons**						
TΔS3675/G3676/F3677	*ORF1a*	CTGCTAGTTGGGTGATGCGTATTATGACATGGTTGGATATGGTTGATACTAGTTTGAAGCTAAAAGA	11,232–11,339	99	MN908947.3	This study
		CTGTGTTATGTATGCATCAGCTGTAGTGTTAC				
TΔH69/V70	*S*	CCTCAGTTTTACATTCAACTCAGGACTTGTTCTTACCTTTCTTTTCCAATGTTACTTGGTTCCATGCTA	21,696–21,816	115	MN908947.3	This study
		TCTCTGGGACCAATGGTACTAAGAGGTTTGATAACCCTGTCCTACC				
TΔE156/F157	*S*	CAATTTTGTAATGATCCATTTTTGGGTGTTTATTACCACAAAAACAACAAAAGTTGGATGGAAAGTG	21,962–22,094	127	MN908947.3	This study
		GAGTTTATTCTAGTGCGAATAATTGCACTTTTGAATATGTCTCTCAGCCTTTTCTTATGG				
TΔΝ211ins214EPE	*S*	GGGAATTTGTGTTTAAGAATATTGATGGTTATTTTAAAATATATTCTAAGCACACGCCTATTATAGTG	22,131–22,277	153	MN908947.3	This study
		CGTGAGCCAGAAGATCTCCCTCAGGGTTTTTCGGCTTTAGAACCATTGGTAGATTTGCCAATAGGTAT				
		TAACATCACTAGGTTTC				
TΔL242/A243/L244	*S*	CTCCCTCAGGGTTTTTCGGCTTTAGAACCATTGGTAGATTTGCCAATAGGTATTAACATCACTAGGTT	22,208–22,342	126	MN908947.3	This study
		TCAAACTTTACATAGAAGTTATTTGACTCCTGGTGATTCTTCTTCAGGTTGGACAGCT				
TIPC	N/A	GGGAATTTGTGTTTAAGAATATTGATGGTTAGCTGCTGTTTACAGTCCAAGATGGTAGTATTCTTGCT	N/A	96	N/A	[36]
		AGTTACACTAGCCATCCTTACTGCGCTT				
**Molecular Beacons** ^f^						
MBΔS3675/G3676/F3677	*ORF1a*	HEX-CGCAGCCCTTTTAGCTTCAAACTAGTATCAACCATATCGGCTGCG-DABCYL	11,267–11,306		MN908947.3	This study
MBΔH69/V70	*S*	HEX-GCCGTCCAGAGATAGCATGGAACCAAGACGGC-DABCYL	21,750–21,777		MN908947.3	This study
MBΔE156/F157	*S*	HEX-CTCGGCGAATAAACTCCACTTTCCATCCAACGCCGAG-DABCYL	22,014–22,044		MN908947.3	This study
MBΔΝ211ins214EPE	*S*	HEX-CACCCCATCTTCTGGCTCACGCACTATGGGGTG-DABCYL	22,193–22,207		MN908947.3	This study
MBΔL242/A243/L244	*S*	HEX-CGGCCTGGTCAAATAACTTCTATGTAAAGTTTGAAACCTAGCAGGCCG-DABCYL	22,269–22,311		MN908947.3	This study
MBIPC	N/A	FAM-GCCCACGTACCATCTTGGACTGTAAACAGCAGCCGTGGGC-DABCYL	N/A		N/A	[36]

^a^ PCR primer, oligonucleotide, and molecular beacon names as these appear in the text; Orientation of the PCR primer is indicated in parenthesis: F, forward; R, reverse. ^b^ Positions/numbering correspond to Wuhan-Hu-1 (GenBank accession number: MN908947.3). In the case of molecular beacons, the positions correspond to the target recognition sequences of the probe. ^c^ Amplicon length denotes the size in nucleotides (nts) of the target amplicons for each of the genes (*S, ORF1a*) as well as the internal positive control (IPC). The primers for IPC are 22,131 (F) and 26,355 (R) [36]. ^d^ The SARS-CoV-2 reference genome Wuhan-Hu-1 (GenBank accession number: MN908947.3) was used for the numbering indicated for the oligonucleotides used in this assay. The identification and isolation of suitable SARS-CoV-2 genomic regions containing the deletions/insertion ORF1a:ΔS3675/G3676/F3677, S:ΔH69/V70, S:ΔE156/F157, S:ΔN211, S:ins214EPE, and S:ΔL242/A243/L244 was performed though the generation of multiple sequence alignments (MSA), using sequences downloaded from GISAID’s database (accessed on 5 July 2021 and 29 November 2021) [59], respective to each VOC, and the SARS-CoV-2 reference genome Wuhan-Hu-1. The design of primers, target amplicons, and molecular beacons was completed using these MSAs. ^e^ The sources of the oligonucleotides used for the real-time RT-PCR assay. ^f^ Underlined regions denote the stem of the molecular beacons; FAM denotes (6-carboxy fluorescein); HEX, (N-HEX-6-Aminohexanol); and DABCYL, (N-[4-(4-dimethylamino) phenylazo] benzoic acid).

**Table 2:** Real-time RT-PCR results of SARS-CoV-2 human clinical samples and reference samples.

Samples ^a^	WHO VOC (Pango Lineage) ^d^	Real-Time RT-PCR Result ^e^				
		ΔS3675/G3676/F3677 or ΔL3674/S3675/G3676	ΔH69/V70	ΔE156/F157	ΔN211ins214EPE	ΔL242/A243/L244
**EVAg ** ^b^						
MT777677.1	B.1	−	−	−	−	−
BetaCoV/Germany/BavPat1/2020 p.1	B.1	−	−	−	−	−
EPI_ISL_918165	Alpha (B.1.1.7)	+	+	−	−	−
EPI_ISL_1834082	Beta (B.1.351)	+	−	−	−	+
EPI_ISL_877769	Gamma (P.1)	+	−	−	−	−
EPI_ISL_2838050	Delta (B.1.617.2)	−	−	+	−	−
**Clinical Samples** ^c^						
BMV2539	Delta (B.1.617.2)	−	−	+	−	−
BMV2543	Delta (B.1.617.2)	−	−	+	−	−
BMV2542	Delta (B.1.617.2)	−	−	+	−	−
BMV5679	Omicron (BA.1)	+	+	−	+	−
BMV4354	Omicron (BA.1.15)	+	+	−	+	−
BMV5890	Omicron (BA.1)	+	+	−	+	−
BMV5872	Omicron (BA.1.17)	+	+	−	+	−
BMV5687	Omicron (BA.1)	+	+	−	+	−
BMV8083	Omicron (BA.2)	+	−	−	−	−
BMV6824	Omicron (BA.2)	+	−	−	−	−
BMV7517	Omicron (BA.2)	+	−	−	−	−
BMV7015	Omicron (BA.2)	+	−	−	−	−
BMV7067	Omicron (BA.2)	+	−	−	−	−
BMV8468	Omicron (BA.4)	+	+	−	−	−
BMV8444	Omicron (BA.4)	+	+	−	−	−
BMV8436	Omicron (BA.4)	+	+	−	−	−
BMV8054	Omicron (BA.4)	+	+	−	−	−
BMV8072	Omicron (BA.4)	+	+	−	−	−
BMV7629	Omicron (BA.5.1)	+	+	−	−	−
BMV8220	Omicron (BE.1.1)	+	+	−	−	−
BMV8173	Omicron (BA.5.8)	+	+	−	−	−
BMV8056	Omicron (BA.5.1)	+	+	−	−	−
BMV8177	Omicron (BA.5.2)	+	+	−	−	−

^a^ The samples used to test the present assay. For the panel of reference samples under “EVAg”, the GenBank and GISAID accession numbers are provided. For the EVAg reference sample BetaCoV/Germany/BavPat1/2020 p.1, only the virus name is provided. For the samples under “Clinical Samples”, the laboratory identification number given by the Laboratory of Biotechnology and Molecular Virology at the University of Cyprus (BMV UCY) is provided. ^b^ The panel of freeze-dried SARS-CoV-2 variants grown in cell culture and the purified RNA of Coronavirus strain BetaCoV/Germany/BavPat1/2020 p.1 grown in cell culture were received from the EVAg (European Virus Archive goes Global) Organization. ^c^ The clinical samples that were selected to be tested by the present assay are part of the study of the Genomic Epidemiology of the SARS-CoV-2 Epidemic in Cyprus ([66] and manuscript in preparation for publication), which were collected under the ongoing collaboration between the BMV UCY and the Ministry of Health. Bioethical approval was received by the Cyprus National Bioethics Committee (EEBK 21.1.04.43.01) for the use of these samples. To ensure patient anonymity, all the samples received by BMV UCY were double coded to ensure no connection between the samples and the corresponding study subjects could be made. The collection and use of the samples were in accordance with the relevant guidelines and regulations of the Cyprus National Bioethics Committee. ^d^ SARS-CoV-2 Variants of Concern (VOC), Alpha, Beta, Gamma, Delta, and Omicron, are as denoted by the World Health Organization (WHO) (WHO Greek alphabet nomenclature). The lineages (and sublineages) in parentheses are based on the Pangolin-lineage (Pango Lineage) SARS-CoV-2 classification system. The B.1 lineage is a direct descendant of the B lineage, which was the original SARS-CoV-2 lineage that was discovered first at the start of the pandemic. ^e^ The assay targets specific deletions and one insertion present across the VOC lineages: ORF1a:ΔS3675/G3676/F3677, S:ΔH69/V70, S:ΔE156/F157, S:ΔN211ins214EPE, and S:ΔL242/A243/L244 (https://covariants.org/ date last accessed 1 December 2022) [55]. Detectable real-time RT-PCR amplification signal (C_T_ values ≤ 40), a positive result, indicating the presence of the targeted deletion assayed in this experiment, is denoted by the symbol (+). Undetectable signal (C_T_ values > 40), a negative result, indicating the absence of the targeted deletion assayed in this experiment, is denoted by (−).

## Data Availability

The results will be available upon request from the corresponding author.

## Supplementary Materials

The following are available online at https://www.mdpi.com/article/10.3390/life13020304/s1, Table S1: Real-time RT-PCR threshold cycle (Ct) results of SARS-CoV-2 human clinical samples and reference samples.
