# Supplementary material for: Detection of Circulating SARS-CoV-2 Variants of Concern (VOCs) Using a Multiallelic Spectral Genotyping Assay
Source: Life (Basel). 2023 Jan 21;13(2):304. doi: 10.3390/life13020304 (PMC9960118; doi:10.3390/life13020304)
Supplement: Supplementary file 1 [file life-13-00304-s001.zip › life-2139641-supplementary.pdf]

Table S1. Real-time RT-PCR threshold cycle (Ct) results of SARS-CoV-2 human clinical samples and reference samples

Samples[a]	WHO VOC (Pango Lineage)[d]	Real-time RT-PCR Result [e]				
		ΔS3675/G3676/F3677 or ΔL3674/S3675/G3676	ΔH69/V70	ΔE156/F157	ΔN211ins214EPE	ΔL242/A243/L244
**EVAg** [b]						
MT777677.1	B.1	> 40	> 40	> 40	> 40	> 40
BetaCoV/Germany/BavPat1/2020 p.1	B.1	> 40	> 40	> 40	> 40	> 40
EPI_ISL_918165	Alpha (B.1.1.7)	17.3	17.0	> 40	> 40	> 40
EPI_ISL_1834082	Beta (B.1.351)	16.8	> 40	> 40	> 40	20.3
EPI_ISL_877769	Gamma (P.1)	17.1	> 40	> 40	> 40	> 40
EPI_ISL_2838050	Delta (B.1.617.2)	> 40	> 40	19.6	> 40	> 40
**Clinical Samples**[c]						
BMV2539	Delta (B.1.617.2)	> 40	> 40	36.0	> 40	> 40
BMV2543	Delta (B.1.617.2)	> 40	> 40	34.3	> 40	> 40
BMV2542	Delta (B.1.617.2)	> 40	> 40	39.5	> 40	> 40
BMV5679	Omicron (BA.1)	32.0	33.6	> 40	37.2	> 40
BMV4354	Omicron (BA.1.15)	28.2	30.8	> 40	32.7	> 40
BMV5890	Omicron (BA.1)	24.5	28.3	> 40	31.9	> 40
BMV5872	Omicron (BA.1.17)	28.7	32.1	> 40	38.1	> 40
BMV5687	Omicron (BA.1)	24.9	27.5	> 40	32.4	> 40
BMV8083	Omicron (BA.2)	30.8	> 40	> 40	> 40	> 40
BMV6824	Omicron (BA.2)	25.1	> 40	> 40	> 40	> 40
BMV7517	Omicron (BA.2)	24.3	> 40	> 40	> 40	> 40
BMV7015	Omicron (BA.2)	30.1	> 40	> 40	> 40	> 40
BMV7067	Omicron (BA.2)	26.7	> 40	> 40	> 40	> 40
BMV8468	Omicron (BA.4)	27.1	30.5	> 40	> 40	> 40
BMV8444	Omicron (BA.4)	23.7	23.2	> 40	> 40	> 40
BMV8436	Omicron (BA.4)	34.4	34.2	> 40	> 40	> 40
BMV8054	Omicron (BA.4)	32.1	31.5	> 40	> 40	> 40
BMV8072	Omicron (BA.4)	28.4	28.3	> 40	> 40	> 40
BMV7629	Omicron (BA.5.1)	27.5	26.9	> 40	> 40	> 40
BMV8220	Omicron (BE.1.1)	29.0	28.1	> 40	> 40	> 40
BMV8173	Omicron (BA.5.8)	26.2	25.0	> 40	> 40	> 40
BMV8056	Omicron (BA.5.1)	29.2	30.4	> 40	> 40	> 40
BMV8177	Omicron (BA.5.2)	34.3	33.6	> 40	> 40	> 40

[a] The samples used to test the present assay. For the panel of reference samples under “EVAg”, the GenBank and GISAID accession numbers are provided. For the EVAg reference sample BetaCoV/Germany/BavPat1/2020 p.1, only the virus name is provided. For the samples under “Clinical Samples”, the laboratory identification number given by the Laboratory of Biotechnology and Molecular Virology at the University of Cyprus (BMV UCY) is provided.

[b] The panel of freeze-dried SARS-CoV-2 variants grown in cell culture, and the purified RNA of Coronavirus strain BetaCoV/Germany/BavPat1/2020 p.1 grown in cell culture, received from the EVAg (European Virus Archive goes Global) Organization.

[c] The clinical samples that were selected to be tested by the present assay are part of the study of the Genomic Epidemiology of the SARS-CoV-2 Epidemic in Cyprus ([66] and manuscript in preparation for publication), which were collected under the ongoing collaboration between the BMV UCY and the Ministry of Health. Bioethical approval was received by the Cyprus National Bioethics Committee (EEBK 21.1.04.43.01) for the use of these samples. To ensure patient anonymity, all the samples received by BMV UCY were double coded to ensure no connection between the samples and the corresponding study subjects could be made. The collection and use of the samples were in accordance with the relevant guidelines and regulations of the Cyprus National Bioethics Committee.

[d] SARS-CoV-2 Variants of Concern (VOC), Alpha, Beta, Gamma, Delta and Omicron are as denoted by the World Health Organization (WHO) (WHO Greek alphabet nomenclature). The lineages (and sublineages) in the parenthesis are based on the Pangolin-lineage (Pango Lineage) SARS-CoV-2 classification system. The B.1 lineage is a direct descendant of the B lineage, which was the original SARS-CoV-2 lineage that was discovered first at the start of the pandemic.

[e] The assay targets specific deletions and one insertion present across the VOC lineages: *ORF1a*:ΔS3675/G3676/F3677, *S*:ΔH69/V70, *S*:ΔE156/F157, *S*:ΔN211ins214EPE and *S*:ΔL242/A243/L244 (https://covariants.org/ date last accessed 01 December 2022) [55]. Detectable real-time RT-PCR amplification signal (C_T values ≤ 40), a positive result, indicating the presence of the targeted deletion assayed in this experiment, is denoted by the threshold cycle (C_T values). Undetectable signal (C_T values > 40), a negative result, indicating the absence of the targeted deletion assayed in this experiment, is denoted by (> 40).
